# Second Wave of COVID-19 in Bangladesh: An Integrated and Coordinated Set of Actions Is Crucial to Tackle Current Upsurge of Cases and Deaths

**DOI:** 10.3389/fpubh.2021.699918

**Published:** 2021-08-30

**Authors:** Razmin Bari, Farhana Sultana

**Affiliations:** ^1^University of North Carolina at Chapel Hill, Chapel Hill, NC, United States; ^2^Department of Political Science and Sociology, North South University, Dhaka, Bangladesh

**Keywords:** COVID-19, Bangladesh, second wave, vaccination, education

Bangladesh had successfully managed to tackle the first wave of coronavirus infections in 2020. However, at present the infection rate has sharply risen to 21–24% and the country is facing the deadliest episode of the outbreak. Each day the infected cases and death counts are reaching new peaks. During mid-January to first week of March, 2021 the infection rate was below 5 and since the last week of March 2021, the infection and death toll gradually increased suggesting the start of the second wave of COVID-19 (Figures 1A,B) (1).

**Figure 1:**
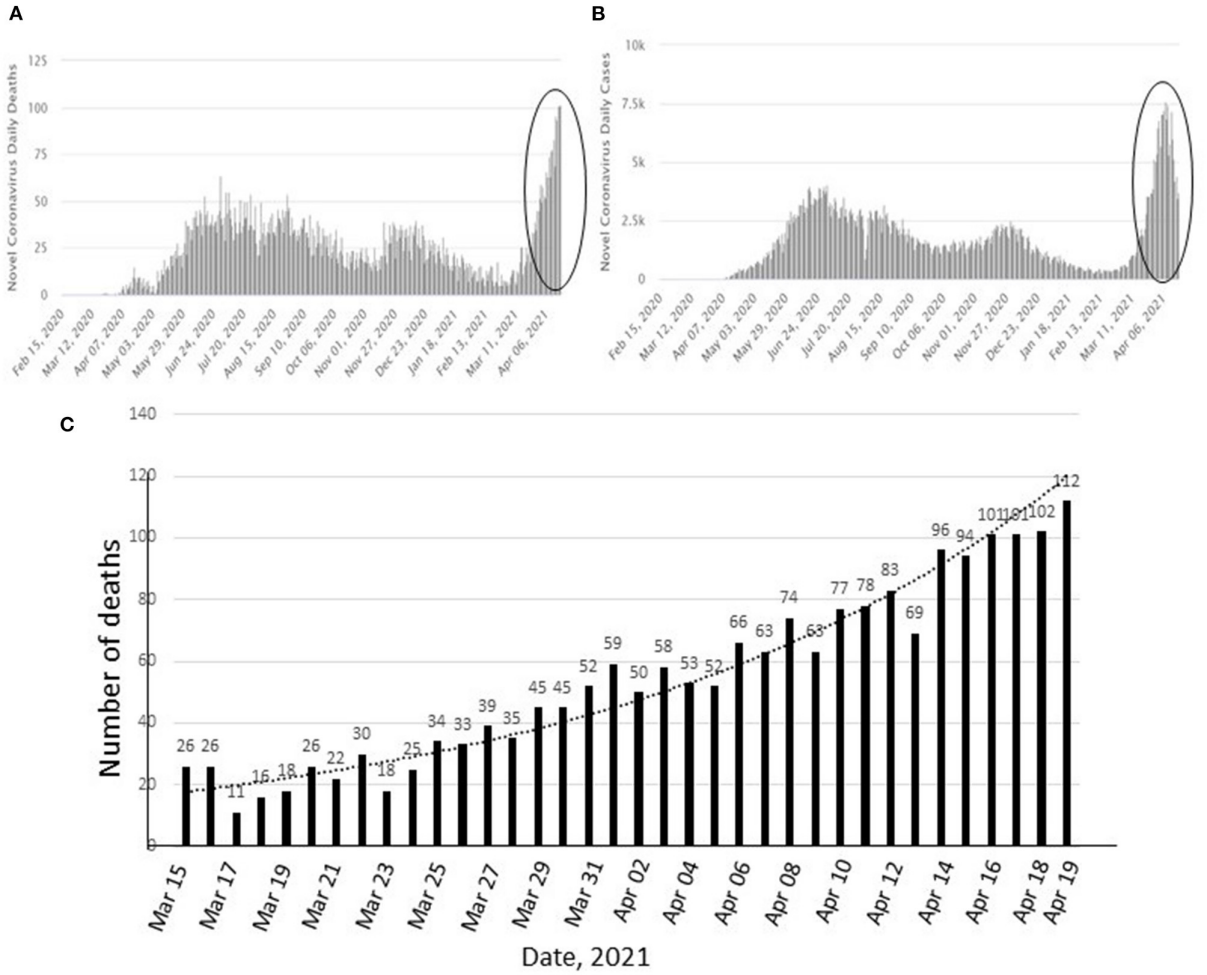
Changes in positive cases and deaths by COVID-19 in Bangladesh since March 2020 to April 19, 2021 **(A,B)**. Circles show the recent upsurge. Increase in number of deaths during March 15 to April 19, 2021 **(C)**. **(A,B)**: source: https://covid19.who.int/region/searo/country/bd, (accessed April 19, 2021).

## Current COVID-19 Scenario

### Pattern of Current Infection, Death, and New Variants

In the past 30 days, the number of coronavirus cases have tripled in Bangladesh. All COVID-19 hospitals in the capital and other cities are completely full with patients; the dedicated beds in many other hospitals are also full. From February 12 to March 13, 2021, 461 patients had died; within the next 30 days, the total death toll rose to 1,263 (1). On March 15, 2021, the number of deaths was only 26 whereas after 1 month, on April 19, 2021, the death toll reached 112, the highest since the start of the pandemic (Figure 1C).

As of April 19, 2021, 723,221 cases have been recorded while 10,497 people have died of COVID-19 in the country (2). About 54% of the infected people are in the age group of 21–40 years, however, the low death rate of 6.92% (3) strongly suggests that people of this age group can fight the virus with their existing immunity (4). More than 80.83% of the deceased were aged above 50 at the time of death; this may be correlated to weak immunity and comorbidities.

The South African variant is speculated to be one of the causes of the recent spike in number of cases and deaths as it is 50% more transmissible than wild-type SARS-CoV-2 (5). A very recent study has demonstrated that more than 80% of current infections is caused by the South African variant (6). It should be noted that the sample size for this study was quite low; hence this study cannot be used to represent the total population and further research is required. Earlier in 2021, the UK variant had also been detected among the Bangladeshi population which has been assumed to trigger the current rise in infection rate as well. Until now, only about 700 genome sequences have been completed in Bangladesh. It is important to continue sequencing coronavirus genomes periodically to understand any significant changes that may cause the emergence of any new deadly variant.

### Factors Deteriorating the Situation Further

Several other factors such as careless attitude of the people toward the virus, not wearing masks and not maintaining physical distancing are aggravating the present situation (7–9). A large fraction of people, mostly residing in villages and slums, show major hesitancy toward vaccination, primarily due to lack of knowledge (10). Anti-vaccine proponents, perception of having low risks of infection, and concerns about adverse events, toxicity, and the overall efficacy of vaccines strongly discourage people from the procedure (11). Some are also apprehensive about the vaccine's long-term effects. Moreover, until very recently, the government also allowed all public and private offices, court, restaurant, shopping malls, recreation centers, religious centers etc. to carry out regular activities without proper monitoring. Unsurprisingly everybody had started to think the country was on the brink of bidding farewell to the coronavirus. But the hard reality is totally opposite.

### Poor Hospital Management

Since the start of the pandemic, huge irregularities in hospital management have been identified (12). The government tried to address some issues, and some high officials were also penalized; but no significant correction in the management system has been evident. Even after a year, the same level of inadequacy in the preparedness to combat COVID-19 is distinctly visible. Several temporary hospitals, quarantine centers, isolation centers had been built which were soon dismantled or turned into general hospitals due to lack of foresightedness and careful planning after the number of cases dropped significantly during November 2020—February 2021.

It is important to note that in most cases of recent infection, the oxygen saturation level falls drastically and patients need immediate intervention with high-flow oxygen nasal cannula or ventilator. A lot of them further require ICU support and when such treatment is not provided timely, elderly people (>50 years) cannot survive and in fact, are dying. The country lacks sufficient oxygen manufacturing plants; the oxygen demand at present has risen to a peak where it is nearly 10 times the supply. Most private hospitals are in huge shortage of oxygen supply, and even the COVID-19 specialized hospitals do not have continuous oxygen supply facility—leading to many deaths that could have been prevented (13).

All general beds and ICU beds for COVID-19 patients across the country are occupied, but unfortunately there are many more infected people in need of emergency medical attention. It is regretful that some patients are dying in the ambulances parked in front of hospitals due to lack of beds. According to IEDCR, in all public and private hospitals in Dhaka city, there are only a total of 4,286 general beds and 499 ICU beds for COVID-19 patients (1). At this moment, twenty-five to thirty patients are in the waitlist for an ICU bed. An additional serious issue is the lack of experts who can provide optimum service as most doctors, physicians and other healthcare professionals do not have working experience in ICU. In response, the government has more recently decided to recruit 2,000 doctors and 3,000 other health professionals to provide service to the infected patients (14). While both general and ICU bed numbers have been increased, it is still insufficient to meet the current demand.

### Insufficient Testing

Both governmental and non-governmental initiatives to motivate people for testing have largely been unsuccessful. People are not interested in testing because it is costly and time-consuming, and because they are not trustful of the test results. Also, there is a large number of asymptomatic cases who are not aware that they require testing. Hence, we have been seeing a downtrend in testing for quite some time (1). In spite of this, testing needs to be increased in order to isolate positive cases and protect other healthy people. At the same time, contact-tracing followed by necessary quarantining and isolation has to be done to contain the disease and break the transmission chain.

Of late, Bangladesh government has set up some new RT-PCR testing centers and also approved antigen testing at low cost at the district level (15); yet, the outcome is not significant as a lot of people are unaware of these facilities and as the test reports take an extended period of time to be delivered due to shortage of trained manpower. Local pharmacy shops may operate as antigen testing centers so that asymptomatic or suspected COVID-19 patients can easily get tested, and timely quarantine measures can be adopted.

Additionally, community-level serological surveillance can be undertaken by mass-testing the population of each community to understand the extent of SARS-CoV-2 infections, including asymptomatic cases. This information is necessary to implement effective public health efforts such as vaccination on priority basis.

Furthermore, wastewater based epidemiological (WBE) surveillance using sewage samples can be implemented to detect genetic materials of the SARS-CoV-2 in densely populated urban areas (16, 17). This will essentially help identify communities with positive cases where mass-testing is not possible, and aid in adopting timely public health interventions to mitigate further spread of the disease.

## Mitigation Measures

### Use of Mask

Under government directives, all public and private offices, banks, restaurants, shopping malls, public transports etc. were already implementing a “No mask, no service” rule sub-optimally since last year. Yet, there are people who still do not care or understand the importance of masks: for example, being driven by religious belief, people visiting mosques often remain mask-less.

### Vaccination

Bangladesh was one of the few countries that started vaccine rollouts timely (18). As of April 19, 2021, 5.73 million people have received at least one dose of the Oxford-AstraZeneca vaccine and 1.51 million people are fully vaccinated with two doses (0.8% population fully vaccinated) (19). Indian Serum Institute had approved the provision of 30 million doses of the Oxford-AstraZeneca vaccine through a tri-partite agreement. Until now, Bangladesh has received only 7 million doses under this agreement, and an additional 3.2 million doses as a gift from India. The country has yet to receive any vaccine via the COVAX initiative despite their commitment to provide 68 million doses. Due to scarcity, the vaccination program is moving at a very slow pace (20). The latest action plan covers only those who have received the first dose; practically no new individual is being encouraged toward vaccination at this moment. Bangladesh needs to work out desperately to procure vaccine for mass people in order to safely open up all activities. It has become especially essential to protect the population of nearly 25 million living in Dhaka and its adjacent areas and Chittagong. It is worth noting that the age limit for vaccination has been lowered to accommodate a greater portion of the population—unfortunately, since there is a shortage of vaccines, this decision may cause negative repercussion as the more vulnerable, elderly people will not receive vaccines on a priority basis anymore.

### Lockdown

After observing the rise in daily cases and deaths, the government had effected the first nationwide lockdown for 10 days, although most of the rules of lockdown were not enforced. A stricter 8-day lockdown period with stringent controlling measures started on April 14, 2021, which has then been extended for 7 more days until April 28 in response to new peaks in death counts everyday. Only essential services as identified by the government have remained open and policing has been strengthened on the streets to stop unwanted movement of the people and vehicles.

With the assurance from the ready-made garments owner to comply 100% with government-directed health protocols, garment workers are also excluded from the lockdown and will continue working in the factories. In reality, many garment owners have not arranged special transportation for their workers. The workers have to find alternative ways to attend work and hence do not end up following health directives. Since the basic needs such as food and medicines are not supplied to the needy people, daily wage-earners and slum dwellers, they are coming out as usual and looking for activities to earn their livelihood, ignoring the government directives.

## Unexpected Movement of People Hampers the Lockdown Outcomes

When the stricter lockdown was announced a few days prior to enforcement, majority of the daily workers, rickshaw-pullers, and private job holders managed to leave Dhaka for their hometowns using rental cars, trucks, motorbikes, speedboats etc. since long-route transportation had already been prohibited (21, 22). These people did not abide by health protocols and have likely carried and transmitted the virus to other dwellers in their villages. The same people are predicted to rush back to the city at the end of the lockdown. All this traveling may lead to a further rise in infections in the currently less-affected areas.

## Closure of Educational Institutions—A Hard-Hit Sector

In response to COVID-19, all schools, colleges and universities has remained closed since March 2020 (23). As an alternative method, online education has been launched, but only an insignificant portion of students in urban areas can avail this opportunity. It is not feasible in rural areas because of multiple problems such as lack of internet coverage, insufficient bandwidth, lack of smart phones, computers gadgets etc.

Students are losing motivation toward study and many children are being psychologically affected (24). A recent study by World Vision demonstrates that 44% of junior-level students are afraid that they might not be able to come back to classes once school activities resume (24). Early childhood marriage in poor families has increased by 44% in 2020 as compared to the previous year. Social violence has equally amplified. The World Vision study further showed that 55% of children are unhappy about their life and staying at home, and 40% of children suffer from malnutrition as the income of their parents had plunged (24).

Due to lack of on-campus activities, all types of hands-on learning and skill development programs are totally absent, and students are receiving a substandard education. The nation is likely to suffer from an insufficiency of skilled and competent graduates as well as professionals to render better service. By vaccinating all students and faculty members on a priority basis and by implementing health protocols, on-campus educational activities should be resumed. The government is seriously contemplating this issue.

## Conclusions

Assuredly, Bangladesh is experiencing a critical time in the second wave of pandemic. The current trend of COVID-19 infection indicates that the world may have to acclimate with the virus for quite a long time. Bangladesh being one of the most densely populated countries with huge working-age population should be more cautious in regards to the COVID-19 pandemic, putting the highest priority to save its precious human resource (25). Otherwise the country's economy and the current GDP growth rate will plummet and people will have to face even more severe hardships. We strongly recommend the following points to be implemented in order to tackle and revert the pandemic situation: (a) Hospital treatment facilities, oxygen supply, ICU beds, high-flow oxygen nasal cannula, and ventilators have to be increased and reserved for case upsurge. (b) More healthcare professionals including doctors and nurses should be trained to provide optimum medical services to critical COVID-19 patients. (c) Based on infection rate, regional lock-down or curfew as a next-level stringent measure for limited time can be imposed. (d) Food and medical needs must be supplied from the government for the people in the lockdown area. (e) More effective, community-engaging awareness campaigns should be organized. (f) Community based surveillance teams should be put to work to diligently help and control residents in following the health directives. (g) Continuous surveying and monitoring need to be conducted to identify the pattern of transmission and carry out contact tracing which is essential for containment. (h) Serological and WBE surveillance should be initiated to identify infected areas for subsequent implementation of containment measures. And finally, (i) to stop the entry of the deadly Delta variant among Bangladesh's residents (26) rigid cautionary measures should be taken at international airports and land ports. All these activities should be performed under a coordinated and integrated national strategic plan.

## Author Contributions

FS conceived the study. FS and RB wrote the manuscript. All authors approved the final version of the manuscript.

## Conflict of Interest

The authors declare that the research was conducted in the absence of any commercial or financial relationships that could be construed as a potential conflict of interest.

## Publisher's Note

All claims expressed in this article are solely those of the authors and do not necessarily represent those of their affiliated organizations, or those of the publisher, the editors and the reviewers. Any product that may be evaluated in this article, or claim that may be made by its manufacturer, is not guaranteed or endorsed by the publisher.
